# Suboptimal nutritional status of school-age children in Addis Ababa: evidence from the analysis of socioeconomic, environmental, and behavioral factors

**DOI:** 10.3389/fpubh.2024.1409202

**Published:** 2024-11-29

**Authors:** Yimer Mihretie Adugna, Abebe Ayelign, Taddese Alemu Zerfu

**Affiliations:** ^1^Center for Food Science and Nutrition, Addis Ababa University, Addis Ababa, Ethiopia; ^2^International Food Research Institute (IFPRI), Addis Ababa, Ethiopia

**Keywords:** Addis Ababa, dietary factors, nutritional status, school-age children, socioeconomic status

## Abstract

**Background:**

School-aged children (6–14 years old) are susceptible to malnutrition and micronutrient deficiencies. Environmental and behavioral factors greatly influence their nutritional status. This study aimed to examine the association between dietary factors and the nutritional status of school-aged children attending public and private schools in Addis Ababa.

**Methods:**

A community-based cross-sectional study design was employed from March to August 2023. A total of 309 study participants were randomly selected from 10 schools. Data were entered into Epidata version 3.1 and exported to SPSS version 23.0 for analysis. WHO Anthro Plus 1.0.4 was used to assess the measurements of weight-for-age (WAZ), height-for-age (HAZ), and BMI-for-age (BAZ) for overweight, stunting, and underweight, while wasting was assessed using MUAC. A Poisson regression model was used to determine the association between predictor variables and stunting, wasting, underweight, and overweight, with adjusted prevalence ratios (APR). APR and 95% CI were used to identify statistically significant variables.

**Results:**

Findings revealed the prevalence of wasting (15%), stunting (24%), underweight (36%), and overweight (19%) among school-aged children. Factors influencing stunting include marital status, house ownership, education level of parents/caregivers, child sex, and meal skipping. Wasting was linked to wealth index, child age, dietary diversity, dietary habits, water access, and toilet facilities. Underweight predictors include parent/caregiver age, marital status, and meal frequency. Moreover, school type appeared as a significant factor for overweight.

**Conclusions:**

The overall nutritional status of school-aged children is suboptimal, influenced by sociodemographic, environmental, and behavioral factors. Addressing these factors through targeted interventions is crucial, particularly for the most vulnerable groups.

## 1 Introduction

Childhood malnutrition remains a pressing global health crisis, particularly in urban low-income settings where complex socioeconomic, environmental, and cultural factors significantly impact dietary habits and contribute to a multifaceted issue affecting millions worldwide (1).

According to the World Health Organization (WHO), malnutrition encompasses deficiencies, excesses, or imbalances in energy and nutrient intake, leading to undernutrition, overweight, overweight and obesity, or micronutrient-related issues (2). Malnutrition affects many people worldwide, with particularly severe consequences in low-income settings (3). In 2023, United Nations International Children's Emergency Fund (UNICEF), World Health Organization (WHO), and the World Bank reported the existence of 148 million stunted, 45 million wasted, and 39 million overweight children around the globe (4). These numbers highlight the urgent need to address malnutrition.

The risk of malnutrition in urban low-income settings increases as there is a high population density, environmental pollution, social marginalization, and violence (5). Also, the abundance of processed, energy-dense foods which lack essential nutrients, contribute to malnutrition problems (6). In urban areas, income, disparities within and between communities highlight the prevalent social and economic inequalities, particularly for the urban poor who dwell in informal settlements or slums (7). The coexistence of both under nutrition and over nutrition shows the complexity of interplay between socioeconomic factors and malnutrition (8). The complex nature of the interaction between malnutrition and socioeconomic factors such as education and income greatly affect women and children. For instance, the households with high level of education and income could have better health knowledge and access to nutritious foods when we compare them to the households with low levels of education and income who could have a high risk of food insecurity and infections (9).

In recent years, Ethiopia has been challenged by a widespread malnutrition crisis which has been exacerbated by conflicts, climate change, and the COVID-19 pandemic. In 2023, it was reported that 31.4 million people required humanitarian aid, including 16.5 million children, with a 19% rise in severely malnourished children (10). School-aged children in Ethiopia experience high rates of stunting, underweight, and wasting compared to the broader African context. Particularly, in Addis Ababa, there were high rates of malnutrition which was a result of a high prevalence of poverty, food insecurity, limited dietary diversity, and susceptibility to diseases (11).

Low-income households are challenged by stunting, wasting, and underweight due to a lack of foods while high-income households are affected by overweight or obesity due to high consumption of processed food (12). As many low-income families cannot afford nutritious foods, they rely on micronutrient-lacking staples, which intensify health risks for (10).

The Ethiopian government, in collaboration with UNICEF and the World Food Programme (WFP), has been attempting to reduce stunting among under-five children by targeting interventions in vulnerable districts (13). Despite their attempt, socioeconomic disparities and the malnutrition issues among the communities in urban poor areas of Addis Ababa persist. This could affect the school-aged children who live in the area too. Therefore, this study aimed to examine the association between dietary factors and nutritional status among school-aged children in urban poor areas of Addis Ababa to provide insights for targeted interventions and policy formulation.

## 2 Materials and methods

### 2.1 Study settings and population

The study was conducted in Addis Ababa, the capital city of Ethiopia. It has an estimated total population of 3,945,000, with a high population density of around 5,165 individuals per square kilometer across its 527 km^2^ (14). Nearly a quarter of Ethiopia's urban population resides in Addis Ababa. The city is administratively divided into 11 sub-cities and 120 districts. The study areas were chosen based on its diversity regarding the socioeconomic landscape of Addis Ababa. Peripheral areas such as Kolfe Keraniyo and Nifas Silk Lafto sub-cities were selected to capture locations characterized by poor infrastructure and housing conditions (15). In addition, inner-city areas, which have modern housing, well-developed road infrastructure, and access to essential amenities, thus catering to high-income individuals, were also included in the study.

### 2.2 Study design

From March 2023 to August 2023, a community-based cross-sectional study was conducted, targeting a cohort of 309 school-aged children in urban low-income settings in Addis Ababa. The study specifically focused on children aged 6–14 years who were enrolled in elementary school (grades 1–8) across 10 districts in two sub-cities: Kolfe Keraniyo and Nifas Silk Lafto. From Kolfe Keraniyo, five districts were included, with each district (referred to as “Woreda”) containing one school: Woreda 04—Jemo No. 1 Primary School, Woreda 05—Addis Hiwot Academy, Woreda 06—Yemane Birhan Academy, Woreda 07—Betel Academy, and Woreda 09—Tinbite Eremyas. Similarly, from Nifas Silk Lafto, five districts were included, each with one school: Woreda 05—Gofa Primary School, Woreda 08—Sibiste Nagash, Woreda 09—Addis Amba Academy, Woreda 13—Abay Academy, and Woreda 15—Hidase Academy. The students' roster was used to select participants from all grades within the study cohort.

### 2.3 Inclusion and exclusion criteria

The study included households that met specific criteria. The households that had at least one child between the ages of six and fourteen, who attended school, and living in the two sub-cities, and the parents or caregivers who signed the consent form to confirm their willingness to participate in this study were included.

### 2.4 Ethics

The study procedures were approved by the Institutional Review Board (IRB) of the College of Natural and Computational Sciences at Addis Ababa University (AAU) (with the code of approval: CNCSDO/515/15/2023). This research protocol adhered to the Ethical Principles for Medical Research Involving Human Subjects as outlined in the Helsinki Declaration amended in Fortaleza, Brazil, in October 2013 (16). All study participants and their guardians were informed to read and sign a voluntary consent form.

### 2.5 Sampling and sampling procedure

The required sample size was determined using the formula for a single population proportion $n=\frac{{\left(Z\frac{\propto }{2}\right)}^{2}p\left(1-p\right)}{d^{2}}$ (17). The parameters used in the calculation were as follows: a 24% prevalence of underweight among school children in Addis Ababa (18), a *Z*-score of 1.96 for a 95% confidence level, a margin of error of 0.05, and a non-response rate of 10%.


$$\begin{array}{r} n=\frac{{\left(1.96\right)}^{2}0.24\left(1-\;0.24\right)}{{\left(0.05\right)}^{2}}\\ n=280.3\;*\;\left(10\%\;\text{non response rate}\right)*\\ n=308.3\approx 309.\; \end{array}$$

**Figure 1:**
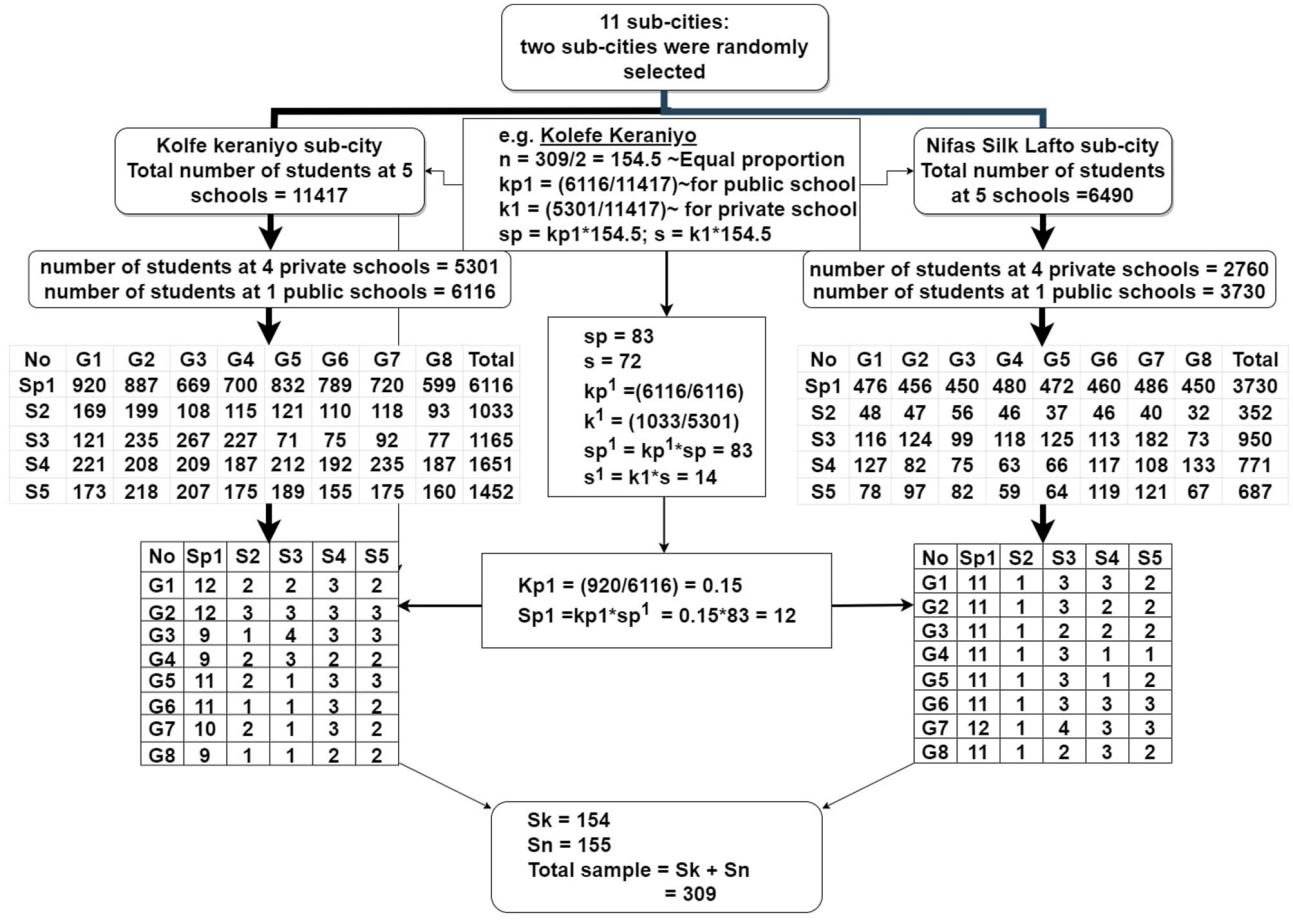
Flow chart of sample selection in urban low-income settings of Addis Ababa. *n*, number of sample calculated; kp1, the ratio of number of students at 1 public school to the total number of students at 5 schools; k1, the ratio of students at four private schools to the total number of students at 5 schools; sp & s, the number of sutdents selected from a public school and private schools; kp^1^, the ratio of number of total primary school students selected at 1 public school to the same total number of students at 1 public school; k^1^, the ratio of total number of students at four private schools to the total number of students at 5 private schools; sp^1^ & s^1^, sample students sleeted from public school and private schools; Kp1, the ratio of students choosen at each grade in public school to the total number of students from (1–8 grade) in 1 school; Sp1, the number of sample students choosen from each grade in the school; Sk & Sn, the final number of students choosen from all 1–8 grade students at both private and public schools from Kolfe Keraniyo and Nifas Silk Lafto sub-cities.

A multi-stage sampling technique using community-based approaches was employed as the sampling strategy. First, the sample size was distributed proportionally to the two sub-cities. Then, five schools were randomly selected from each sub-city using simple random sampling (SRS). Next, a number of school children were systematically sampled from each school by calculating the sampling interval (K). Then, students from (1–8 grade) were randomly chosen from each private and public school using the students' roster, with two public schools and eight private schools (Figure 1). Finally, 309 households were selected based on children's guidance. To assess dietary intake, one child per household was selected. One child was chosen randomly from a household that had more than one school–aged child.

### 2.6 Data collection procedures

In this study, the data were collected in two rounds. The first-round data collection was carried out during a fasting period when followers of the Orthodox religion refrained from consuming animal- source foods. The second-round data collection was carried out after the fasting period, during which all children consumed all types of food. A structured interviewer-administered questionnaire was used to collect the data. The questionnaire encompassed sociodemographic factors, namely, age, sex, gender, education, income, occupation, marital status, household size, religion, household wealth index derived using factorial analysis from a polychoric correlation matrix using a 0.22 cutoff point, grade level of children, and school type. Additionally, it included the following dietary factors: dietary diversity, meal frequency, meal skipping, awareness of nutrition, dietary habits, and absorption inhibitors. Dietary diversity was calculated from the 24-h recall record and categorized into six groups based on the Ethiopian food-based dietary guidelines. Awareness of nutrition was estimated by transforming the two-knowledge-based questions “Which types of foods are included in organic meats? Do you think they are good sources of dietary iron?” and responses (1) beef, lamb, veal, chicken, fish, (2) none, (3) I don't know; and “Which types of foods do you think are good sources of vitamins?” and the responses (1) sweet potato, pumpkin/squash, carrot, kale, papaya, mango, grape, watermelon, liver, egg, milk, (2) none, and (3) I don't know. Then, we assigned one point for a correct answer and 0 points for the incorrect answer according to the FAO (19). Responses falling under category (1) were seen as signs of “awareness,” making up over 50% of correct responses from the respondents. On the other hand, answers in categories (2) and (3), which were < 50% of correct responses from the respondents, were considered as showing a “lack of awareness.”

Dietary habit was also derived from the questions “Do you consume fruits and vegetables daily?” and “Do you usually keep fruits at home and buy lots of vegetables?” and the responses (1) yes, (2) no. Then, based on the number of correct responses from the respondents, more than 50% of the respondents chose yes (1), which transformed into a good habit, and < 50% of them chose (2) and (3), which transformed into no (0) “poor habit.” Meal frequency was determined based on regular mealtime patterns. Children who consumed meals three and more than three times per day, adhering to the typical meal schedule, were categorized as “three and more than three mealtimes.”

Conversely, those who ate meals less than three times per day were classified as “Less than three mealtimes.” Meal skipping was assessed through the question, “How many meals did you skip yesterday?” with responses categorized as (1) one mealtime skipped or less from the usual three mealtimes, classified as “not skipped,” and (2) two or more mealtimes skipped from the usual three mealtimes, classified as “skipped.” The absorption inhibitor was also calculated after the children were asked a question like “Do you drink coffee or tea daily?” and responses (1) “yes,” (2) “no.” This study used sex as a biological attribute to differentiate male and female and gender as social and cultural norms as per the Sex and Gender Equity in Research (SAGER) guideline (20). This guideline helped researchers to ensure that this study would be inclusive and provide insights into potential sex and gender differences.

### 2.7 Wealth index determination

In this study, the asset ownership wealth index was used to determine the economic disparity. A common factor score was assigned to each household using the polychoric correlation matrix. This process included variables that ranged from 0 to 1. Only variables with a cutoff point above 0.22 were used to calculate the factor score. The factor scores were then summed up and divided into three groups of socioeconomic status: poor, medium, and rich. Household assets such as electricity, toilet facilities, televisions, radios, beds, tables, bicycles, motorbikes, refrigerators, and the type of floor material were the factors that determined these groups. The wealth index values were derived from the PCA with the highest eigenvalues and then split into three wealth categories: poor, medium, and rich. This index is a relative measure of household economic status (21).

### 2.8 Dietary diversity assessment

The dietary assessment questionnaire consisted of six food groups: cereals, grains, roots, and tubers; legumes, nuts, and oilseeds; milk and dairy products; meat, fish, and eggs; fruits and vegetables; and fats and oils. These groups, as outlined in the Ethiopian FBDG, were used to assess dietary diversity (22). These groups included cereal, grains, white roots, and tubers; legumes, nuts, and oil seeds; milk and dairy foods, meat, fish, and egg; fruits and vegetables; and fats and oils. To record all food consumption, the 24-h dietary recall method was utilized. Parents/caregivers reported the ingredients and quantities of foods, whether consumed or not, with the aid of photographs for each item to aid in the recall and verification of consumption within the past 24 h. The NutriSurvey software (https://www.nutrisurvey.de/) was employed to calculate the energy and nutrient content based on the 24-h recall protocol (23). Dietary diversity scores (DDS) were calculated from the food groups using a “yes”/“no” response and categorized as high (if four and more than four food groups were consumed) or low (if less than four food groups were consumed), following the Ethiopian FBDG. The adequacy of macronutrient and micronutrient intake was evaluated according to the Dietary Reference Intakes (DRI) set by The Institute of Medicine of The National Academies (24). Reported energy intakes were compared to minimal energy requirements to assess the adequacy.

### 2.9 Anthropometrics indices

Anthropometric measurements were obtained for all school children at their respective schools. The average weight, height, and Mid-Upper Arm Circumference (MUAC) measurements were recorded for each child. The anthropometric indices, such as weight-for-age (WAZ), BMI-for-age (BAZ), and height-for-age (HAZ) standard *Z*-scores, were calculated using the WHO Anthro Plus 1.0.4 software (25). However, the software did not calculate the WAZ for children above 10 years old, resulting in missing data in the SPSS analysis. To address this issue, the MUAC measurement was used to classify the children's nutritional status as either wasting or normal, following the Food and Nutrition Technical Assistance (FANTA) guidelines (26). Children were classified as stunted or underweight when their HAZ and BAZ scores were below −2 SD, and as overweight when their BAZ scores were above +2 SD, respectively.

### 2.10 Statistical analysis methods

Data analyses were conducted using IBM SPSS Statistics version 23 (27). Frequency and percentage were computed for categorical variables. The association between nutritional status and dietary diversity with sociodemographic factors was evaluated separately by a binary logistic regression model in univariate analysis. Following this, adjusted prevalence ratios (APR) in the form of incidence rate ratios (IRR) were estimated using Poisson robust regression in STATA version 16 (28), with a 95% confidence interval (95% CI). A significance level of 5% (*p*-value < 0.05) was applied. Outliers were checked, and multicollinearity was examined using the variance inflation factor (VIF) with a cut-off set below 5.

### 2.11 Data quality control

Three data collectors, one of them as a supervisor, were assigned to collect the data. The questionnaire was initially written in English and subsequently translated into the Amharic language. The translation was carried out by a higher institution English language instructor and a native speaker of Amharic. Data collectors were trained for 2 days focusing on interviewing techniques, questionnaire content, and anthropometric measurements. Several steps were taken to minimize bias during this study. Firstly, the data collectors were not informed about the survey's purpose or content. Similarly, the study's hypothesis was kept hidden from the study respondents to ensure unbiased responses. A pre-test of the study tool was conducted using 10% of the sample from outside the study subjects and made the necessary corrections to address spelling errors and grammar problems based on the pre-test data.

To establish a trusting relationship with the participants, they were encouraged to be transparent about the importance of truthful answers. Clear instructions were provided on how to complete surveys or questionnaires and highlighted the significance of accuracy. To minimize reliance on the participants' memories, questions were designed focusing on recent or significant events.

Lastly, we allowed participants to choose the “Don't Know” or “Prefer Not to Answer” options. A multiple 24-h recall questionnaire including photographs of various food groups and equipment was used to minimize recall bias. Multivariate logistic regression for confounders was used to control for confounding. With these measures in place, the reliability and unbiasedness of the research were enhanced. For data cleaning, Epi-data version 3.1 (29) was used to identify and correct errors, inconsistencies, and anomalies in the data.

### 2.12 Ethical issue

The study was conducted as per the Declaration of Helsinki, and approved by The Institutional Review Board (IRB) of the College of Natural and Computational Sciences of Addis Ababa University (AAU) (code of approval: CNCSDO/515/15/2023, date of approval 20 February 2023).

## 3 Results

### 3.1 Sociodemographic characteristics of caregivers/parents and children

In this study, 309 households participated with response rate of 100%. The sociodemographic characteristics of the parents/caregivers are presented in Table 1. The majority of the parents/caregivers were married (67%), had a diploma or higher education (61.2%), and worked in the private sector (35%). The ages of the parents/caregivers ranged from 18 to 65 years old, with an average age of 33.3 years. On average, the parents/caregivers had a family size of five. Compared to high-income and wealthy caregivers, those with lower incomes and poorer parents were more likely to have larger families, lower education levels, and government-subsidized rent.

**Table 1:** Sociodemographic characteristics of caregivers/parents in urban low-income settings of Addis Ababa, Ethiopia, 2023.

**Background characteristics**	**Categories**	***n* (%)**
Caregivers/parents age	18–25 years	94 (30.4)
	26–35 years	102 (33.0)
	36 and above	113 (36.6)
Gender	Male	146 (47.2)
	Female	163 (52.8)
Family size	2–4 family size	142 (46.0)
	5–8 family size	94 (30.4)
	Above 8 families	73 (23.6)
Marital status	Married	207 (67.0)
	Single	53 (17.2)
	Divorced	29 (9.4)
	Widowed	20 (6.5)
Educational status	Diploma and above	189 (61.2)
	9–12 grade	62 (20.1)
	1–8 grade	23 (7.4)
	Read and write only	18 (5.8)
	Unable to read and write	17 (5.5)
Occupation	Government	72 (23.3)
	Private	108 (35.0)
	Farmer	11 (3.6)
	Merchant	68 (22.0)
	Housewife	50 (16.2)
Household income	Low ( ≤ 7,000 birr)	88 (28.5)
	Middle (8,000–10,000 birr)	78 (25.2)
	High (≥11,000 birr)	143 (46.3)
Religion	Orthodox Christian	141 (45.6)
	Muslim	75 (24.3)
	Protestant	64 (20.7)
	Others^*^	29 (9.4)
House owner	Private Owned	162 (52.4)
	Rent from private	105 (34.0)
	Rent from gov't	42 (13.6)
Wealth index	Poor	85 (27.5)
	Middle	106 (34.3)
	Rich	118 (38.2)

%, percentage of respondent; n, number of respondents.

^*^Catholic, Jehovah, and wake feta.

The sociodemographic characteristics of the school-aged children in their age group were evenly distributed, with 49.8% falling into the 6–10 years category and 50.2% falling into the 11–14 years category. Similarly, the distribution of school type was almost equal, with 55.7% attending public school and 44.3% attending private school. However, as it can be seen in Table 2, there were more females (57.6%) than males (42.4%).

**Table 2:** Sociodemographic characteristics, nutritional status, and dietary diversity score of children in urban low-income settings of Addis Ababa, Ethiopia, 2023.

**Characteristics**	**Categories**	***n* (%)**
Age in years	6–10 years	154 (49.8)
	11–14 years	155 (50.2)
Sex	Male	131 (42.4)
	Female	178 (57.6)
School type	Public school	172 (55.7)
	Private school	137 (44.3)
Children school grade	1–4 Grade	150 (48.5)
	5–8 Grade	159 (51.5)
Meal frequency	Less than three meal times	153 (49.5)
	Three and above 3 meal times	156 (50.5)
Dietary diversity score	< 4 food groups	203 (65.7)
	4 and above food groups	106 (34.3)
Skipping meal	Not skipped	134 (43.4)
	Skipped	175 (56.6)
Dietary habit	Good dietary habit	125 (40.5)
	Poor dietary habit	184 (59.5)
Absorption inhibitor	No	145 (46.9)
	Yes	164 (53.1)
Water supply	No	122 (39.5)
	Yes	187 (60.5)
Toilet	No	173 (56.0)
	Yes	136 (44.0)
Wasting	Wasting	46 (14.9)
Stunted indices	Stunted	75 (24.3)
BMI Indices	Underweight	111 (35.9)
	Overweight	58 (18.8)
Height for age	Mean ± SD	−0.78 ± 1.59
BMI for age	Mean ± SD	−1.03 ± 2.08
MUAC (mm)	Mean ± SD	194 ± 29
Weight (kg)	Mean ± SD	32.1 ± 9.7
Height (cm)	Mean ± SD	139.8 ± 15.3

%, percentage of the respondent; MUAC, mid-upper arm circumference; BMI, body mass index; mean, average value; SD, standard deviation.

More than half (59.5%) of school-aged children had poor dietary habits, as evidenced by meal skipping (56.6%) and the consumption of an undiversified diet (less than four food groups) (65.7%). The mean (±SD) height-for-age, BMI-for-age, MUAC, weight, and height were recorded as −0.78 (±1.59), −1.03 (±2.08), 194 (±29) mm, 32.1 (±9.7) kg, and 139.8 (±15.3) cm, respectively. Furthermore, the prevalence of wasting, stunting, underweight, and overweight among children was 14.9%, 24.3%, 35.9%, and 18.8%, respectively.

More than half of the children had access to a water supply (60.5%) and consumed absorption inhibitors (53.1%). However, less than half had access to toilet facilities (44%) and only 50.5% ate three or more meals per day.

### 3.2 Macro and micronutrient intake of school-age children

**Figure 2:**
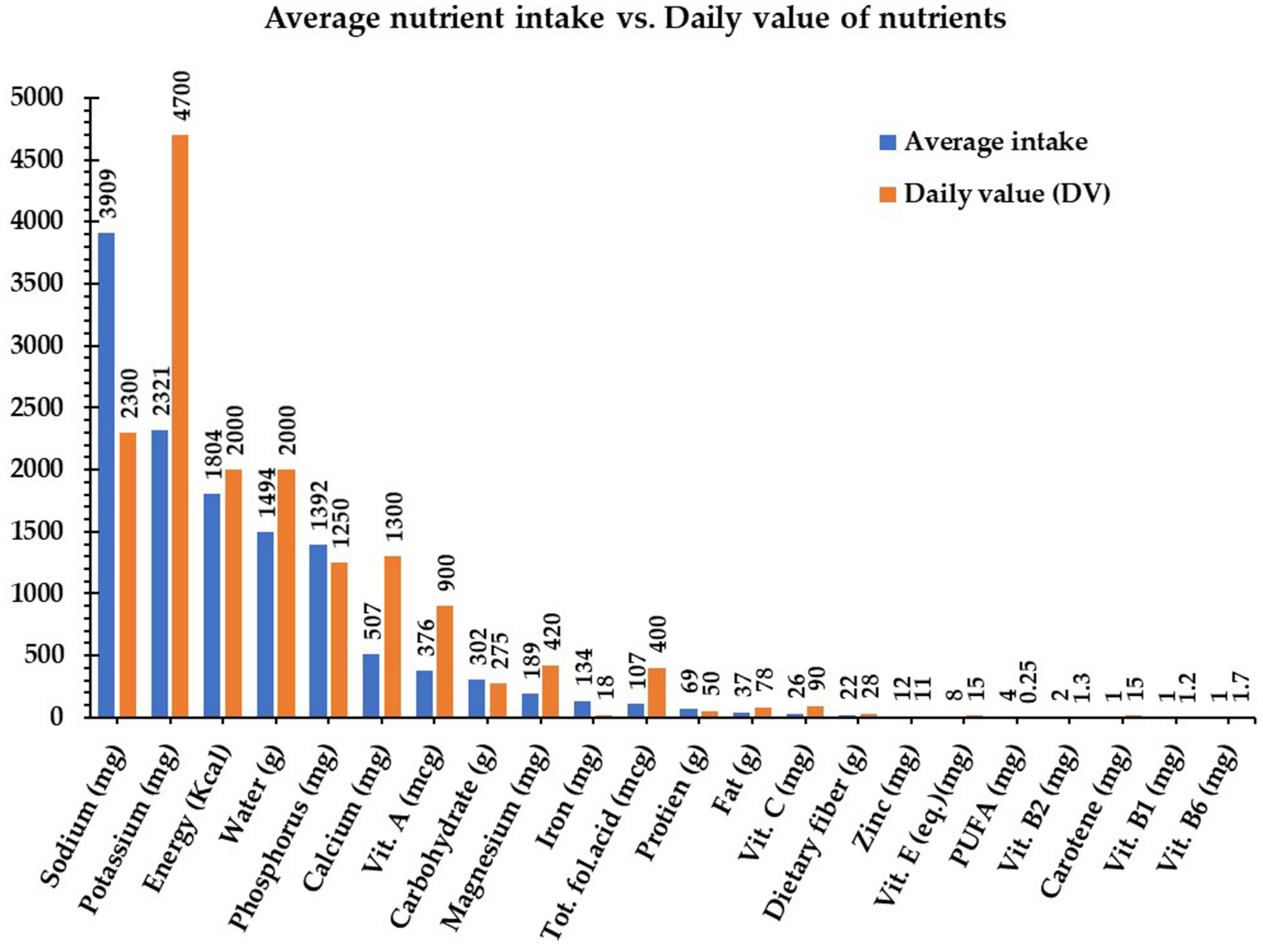
The average intake of nutrients by school-age children vs. the recommended daily value.

The average macronutrient intake for school-aged children fell within the following acceptable ranges: 302.7 g of carbohydrates (45%−65% of energy), 69 g of protein (10%−35% of energy), and 36.8 g of fat (20%−35% of energy). The average energy intake was 1,803.8 kcal, which is slightly below the recommended levels. Micronutrient intake raised the following concerns: calcium at 507.2 mg is below the recommended 1,000 mg, iron at 133.6 mg exceeds the recommended value, and vitamin A at 375.4 μg surpasses the recommended value. However, the intake of polyunsaturated fatty acids was 4.2 g, which is below the adequate daily intake (10.5–17.5 g for boys and 9.1–22.3 g for girls) (30, 31). Additionally, potassium was deficient at 2,322.7 mg compared to the recommended 4,700 mg. The average nutrient intake among school-age children is presented in Figure 2.

### 3.3 Macro- and micronutrient intake by school type

**Figure 3:**
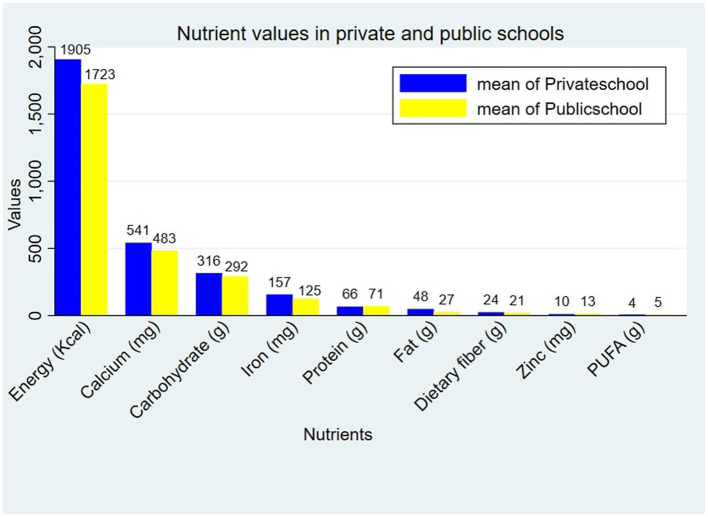
The distribution of nutrient intake within school type.

There is a statistically significant difference in the macro- and micronutrient intake between private- and public-school students. Public school children had a slightly lower mean energy intake level (1,723.39 Kcal) compared to private school children (1,905.91 Kcal; *p* < 0.05). Similarly, the mean fat intake (26.67 g), carbohydrate intake (291.45 g), dietary fiber intake (20.56 g), calcium intake (483.37 mg), and iron intake (124.51 mg) were lower than those of the private school children. However, their mean protein intake (70.59 g), and zinc intake (13.28 mg) were slightly higher than those of the private school children (Figure 3).

### 3.4 Dietary diversity and nutritional gaps in urban low-income school children

**Figure 4:**
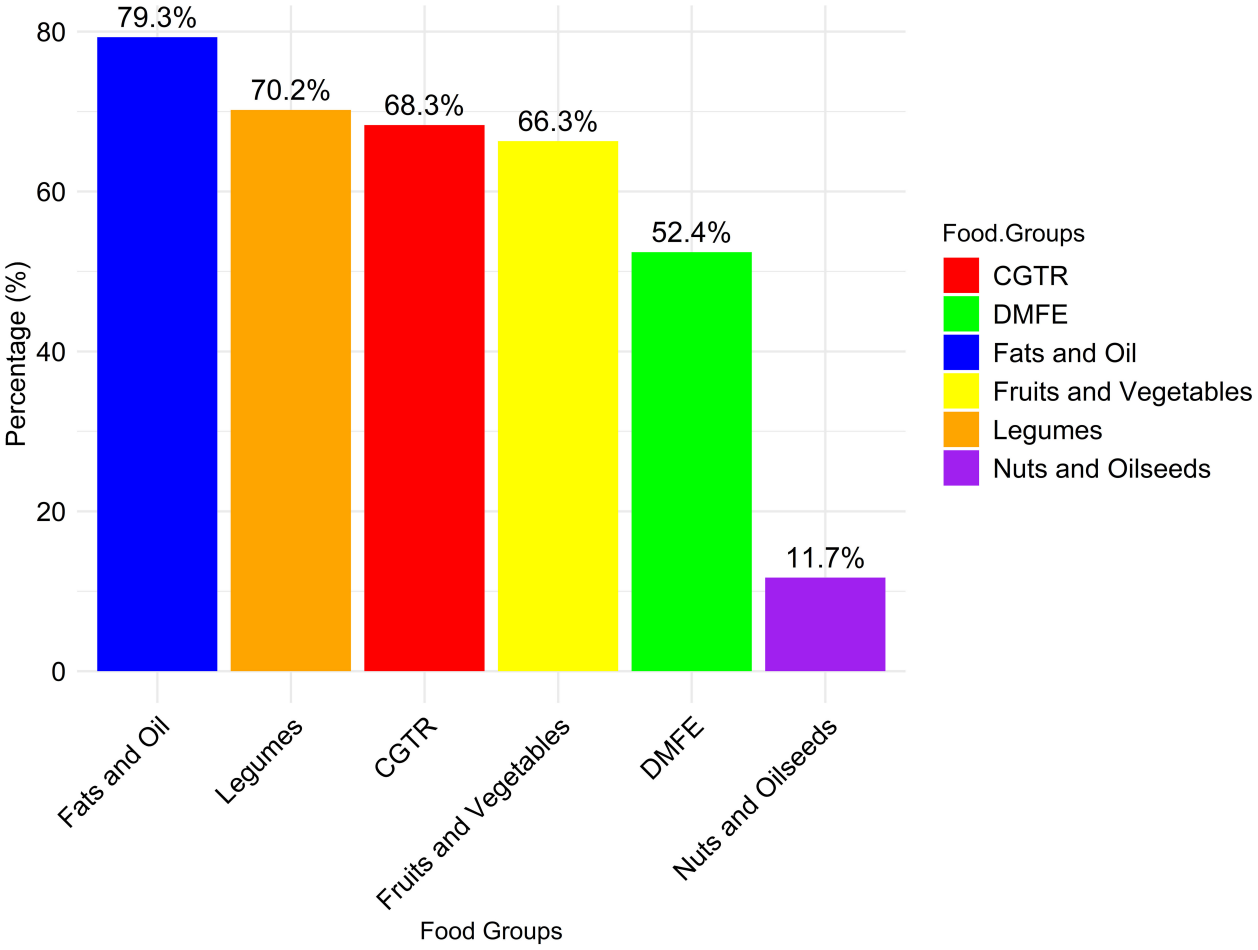
Percentage distribution of food categories consumed by school children in urban low-income areas of Addis Ababa.

In low-income urban areas within Addis Ababa (in Addis Ababa the population is mixed, some are poor and some are rich), school-aged children exhibited distinct food consumption patterns (Figure 4). Generally, school-aged children's diets were dominated by fats and oils, constituting 79.3% of the diet, which reflects a notable reliance on fat sources. The legumes closely followed (70.2%), providing significant protein and fiber. Fruits and vegetables contributed substantially (66.3%), offering essential vitamins and minerals. Cereals, grains, roots, and tubers (CGTR) constituted 68.3%, highlighting their significance. Milk and dairy foods, meat, fish, and eggs (DMFE) accounted for 52.4%, while nuts and oilseeds represented a lower proportion (11.7%). Emphasizing a balanced approach across food groups is essential for a comprehensive nutrient intake. The nutritional gap appears to be related to the consumption of nuts and oil seeds, with a frequency of only 11.7%. This shows a potential deficiency in essential fatty acids and other nutrients in this food group.

### 3.5 Nutritional status of school-age children

The mean (±SD) of BMI-for-age, height-for-age, and MUAC of school-aged children were −1.03 (±2.08), −0.78 (±1.59), and 194 (±29), respectively. According to the WHO growth reference for school-aged children, 24% (95% CI: 20.08–28.46) were stunted, 15% (95% CI: 11.65–18.13) were wasted, 36% (95% CI: 31–41) were underweight, and 19% (95% CI: 15–23) were overweight.

### 3.6 Factors associated with stunting among school-age children

The findings of the adjusted prevalence ratios from Poisson regression, highlighting the variables associated with stunting in school-aged children, are presented in Table 3. The results indicate that children from single-parent households were 2.5 times more likely to experience stunting (APR: 2.5, 95% CI: 1.62, 3.86) compared to those from married households. Additionally, children from divorced households were 2.1 times more likely to be stunted (APR: 2.1, 95% CI: 1.23, 3.54) compared to those with married parents. Renting from private or government sources increased the prevalence of stunting by 2.13 times (APR: 2.13, 95% CI: 1.36, 3.34), and (APR: 2.2, 95% CI: 1.25, 3.76), respectively, compared to private ownership. Lower levels of parental education were strongly associated with increased prevalence of stunting. Children whose parents were able to read and write had 2.7 times higher prevalence of stunting (APR: 2.7, 95% CI: 1.40, 5.11) compared to those with a diploma or higher education. Additionally, female school-aged children had significantly lower prevalence of stunting compared to males (APR: 0.65, 95% CI: 0.0.45, 0.96), while skipping meals increased in the prevalence of stunting 2.03 times (APR: 2.03, 95% CI: 1.33, 3.10). School-aged children whose caregivers lack awareness about nutrition had a 1.5 times higher prevalence of stunting (APR: 1.5, 95% CI: 1.02, 2.16) compared to those whose caregivers or parents are more aware of nutrition.

**Table 3:** A Poisson regression for factors associated with stunting among school-aged children (n = 309) in urban low-income settings of Addis Ababa, Ethiopia, 2023.

**Predictor variables**	**Stunting (height for age) HAZ** < −**2SD**^**a**^			
	**No (%)**	**Yes (%)**	**APR (95% CI)**	***p***<**0.05**
**Marital status**				
Married	174 (74.4)	33 (44.0)	Ref	
Single^**^	29 (12.4)	24 (32.0)	2.5 (1.62, 3.86)	0.000
Divorced^**^	18 (7.7)	11 (14.7)	2.1 (1.23, 3.54)	0.006
Widowed	13 (5.6)	7 (9.3)	1.4 (0.70, 2.61)	0.366
**House owner**				
Private owned	139 (59.4)	23 (30.7)	Ref	
Rent from private^**^	68 (29.1)	37 (49.3)	2.13 (1.36, 3.34)	0.001
Rent from gov't^**^	27 (11.5)	15 (20.0)	2.2 (1.25, 3.76)	0.006
**Education level of parents**				
Diploma and above	161 (68.8)	28 (37.3)	Ref	
9–12 grade^**^	41 (17.5)	21 (28.0)	2 (1.22, 3.27)	0.006
1–8 grade^**^	13 (5.6)	10 (13.3)	2.6 (1.46, 4.48)	0.001
Read and write only^**^	10 (4.3)	8 (10.7)	2.7 (1.40, 5.11)	0.003
Unable to read and write^*^	9 (3.8)	8 (10.7)	1.9 (1.09, 3.30)	0.023
**Child gender**				
Male	87 (37.2)	44 (58.7)	Ref	
Female^*^	147 (62.8)	31 (41.3)	0.65 (0.45, 0.96)	0.031
**Meal skipping**				
Not skipped	115 (49.1)	19 (25.3)	Ref	
Skipped^**^	119 (50.9)	56 (74.7)	2.03 (1.33, 3.10)	0.001
**Information about nutrition**				
Awareness	157 (67.1)	32 (42.7)	Ref	
Lack of awareness^*^	77 (32.9)	43 (57.3)	1.5 (1.02, 2.16)	0.04

^a^Except for the p-value, all figures in the table are rounded to two digits.

APR, adjusted prevalence ratio; CI, confidence interval; HAZ, height for age z-score; Ref, reference category.

^*^p-value < 0.05.

^**^p-value < 0.01.

### 3.7 Factors associated with wasting among school-age children

Table 4 outlines the results of the Poisson regression analysis of the factors linked to wasting in school-aged children. Findings indicate that children from affluent households had significantly lower prevalence of wasting compared to those from poor households (APR: 0.88, 95% CI: 0.80, 0.96). Similarly, children aged 11–14 exhibited lower prevalence of wasting than those aged 6–10 (APR: 0.90, 95% CI: 0.82, 0.98). Lower prevalence of wasting was associated with consuming four or more food groups (APR: 0.86, 95% CI: 0.77, 0.95) and having access to a water supply (APR: 0.84, 95% CI: 0.77, 0.92), while higher prevalence of wasting was associated with poor dietary habits (APR: 1.17, 95% CI: 1.06, 1.29) and the presence of absorption inhibitors (APR: 1.15, 95% CI: 1.05, 1.26).

**Table 4:** A Poisson regression analysis for factors associated with wasting among school-aged children (n = 309) in urban low-income settings of Addis Ababa, Ethiopia, 2023.

**Predictor variables**	**Wasting (MUAC in cm) (*****Z*** < −**2SD)**			
	**No (%)**	**Yes (%)**	**APR (95% CI)**	***p***<**0.05**
**Wealth index (WI)**				
Poor	115 (43.7)	7 (15.2)	Ref	
Middle^**^	78 (29.7)	17 (37.0)	0.88 (0.80, 0.96)	0.007
Rich^**^	70 (26.6)	22 (47.8)	0.82 (0.73, 0.92)	0.001
**Child age**				
6–10 years	140 (53.2)	14 (30.4)	Ref	
11–14 years^*^	123 (46.8)	32 (69.6)	0.90 (0.82, 0.98)	0.02
**Child DDS** ^#^				
< 4 food groups	181 (68.8)	22 (47.8)	Ref	
≥4 and above^**^	82 (31.2)	24 (52.2)	0.86 (0.77, 0.95)	0.004
**Dietary habit**				
Good DH	95 (36.1)	30 (65.2)	Ref	
Poor DH^**^	168 (63.9)	16 (34.8)	1.17 (1.06, 1.29)	0.002
**Absorption inhibitor (AI)**				
No	112 (42.6)	33 (71.7)	Ref	
Yes^**^	151 (57.4)	13 (28.3)	1.15 (1.05, 1.26)	0.002
**Water access**				
No	114 (43.3)	8 (17.4)	Ref	
Yes^**^	149 (56.7)	38 (82.6)	0.84 (0.77, 0.92)	0.000

MUAC, mid-upper arm circumference; cm, centimeter.

^#^Dietary diversity score.

^*^The variable is significant at 0.05 level.

^**^The variable is significant at 0.01 level.

### 3.8 Factors associated with underweight and overweight among school-age children

Table 5 displays the results of a Poisson regression analysis of factors influencing the prevalence of underweight and overweight in school-aged children, with Adjusted Prevalence Ratios (APR) calculated using Poisson robust regression. Findings indicate that children with parents/caregivers aged 26–35 faced a lower underweight risk (APR: 0.57, 95% CI: 0.39, 0.84) compared to those with older caregivers (36 years and above). Children with divorced parents/caregivers had a lower prevalence of underweight (APR: 0.22, 95% CI: 0.07, 0.65). Less than three meals per day correlated with a higher prevalence of underweight (APR: 1.4, 95% CI: 1.04, 1.88), attending public schools reduced the risk of being overweight (APR: 0.44, 95% CI: 0.27, 0.72).

**Table 5:** A Poisson regression analysis for factors associated with underweight and overweight among school-aged children in urban low-income settings of Addis Ababa, Ethiopia, 2023.

**Variables**	**Underweight (*****Z*** < −**2SD)**				**Overweight (*****Z*** > **2SD)**	
	**No (%)**	**Yes (%)**	**APR (95% CI)**	**p**<**0.05**	**APR (95% CI)**	**p**<**0.05**
**Caregivers/parents age**						
18–25 years	56 (28.3)	38 (34.2)	0.99 (0.73, 1.37)	0.987	0.71 (0.36, 1.41)	0.325
26–35 years^**^	77 (38.9)	25 (22.5)	0.57 (0.39, 0.84)	0.005	1.67 (1.01, 2.78)	0.046
36 and above	65 (32.8)	48 (43.2)	Ref		Ref	
**Marital status**						
Married	122 (61.6)	85 (76.6)	0.89 (0.53, 1.49)	0.649	0.57 (0.24, 1.35)	0.201
Single	39 (19.7)	14 (12.6)	0.57 (0.30, 1.10)	0.096	0.96 (0.37, 2.51)	0.939
Divorced^*^	26 (13.1)	3 (2.7)	0.22 (0.07, 0.70)	0.01	1.26 (0.50, 3.16)	0.625
Widowed	11 (5.6)	9 (8.1)	Ref		Ref	
**Meal frequency**						
< 3 meal times^*^	89 (44.9)	64 (57.7)	1.4 (1.04, 1.88)	0.025	1.00 (0.64, 1.59)	0.987
≥3 meal times	109 (55.1)	47 (42.3)	Ref		Ref	
**School type**						
Public school^**^	99 (39.4)	38 (65.5)	1.10 (0.82, 1.48)	0.517	0.44 (0.27, 0.72)	0.001
Private school	152 (60.6)	20 (34.5)			Ref	

SD, standard deviation; Ref, reference category; Z < −2SD, z score below negative 2 standard deviation from the normal (reference category) is undernutrition; Z > 2SD, z score above positive 2 standard deviation from the normal is overweight.

^*^p-value < 0.05.

^**^p-value < 0.01.

## 4 Discussion

The finding, in this study, indicates acceptable macronutrient intake among school- aged children, with a notable reliance on fats and oils, and legumes as the second most consumed food group; however, there is lower consumption of micronutrients, which underscores the importance of achieving a balanced nutrient intake across various food groups. Consumption of diets lower in micronutrients by school-aged children could have an impact on their physical and cognitive development in their later age (32).

The finding, in this study, also reveals nutrient imbalances when compared to recommended values. Low intake of energy, calcium, polyunsaturated fatty acids, and potassium may contribute to stunted growth, weakened bones, and an increased risk of cardiovascular issues (33). Conversely, excessive intake of iron and vitamin A, exceeding recommended levels, could lead to toxicity and other health complications (34).

With regard to stunting, school-aged children in Addis Ababa were found to have stunting with the rate of 24.3%. This signifies that there is still a significant proportion of children in the city who are experiencing growth faltering. In line with this, studies have shown that stunting in school- aged children has a significant impact on their physical growth, cognitive development, and academic performance (35). This study finding shows consistency with a study from Jimma (36) however, this is quite lower than estimates from several other regions in Ethiopia including Arba Minch city (41.9%) (37), Gondar town (46.1%) (38), and Humbo District (57%) (39). On the other hand, it is higher than estimates from Bahir Dar City (15.13%) (36). This variation is likely due to differences in study settings, methods, sample sizes, socioeconomic factors, dietary practices, environmental conditions, and healthcare services among the diverse communities. Therefore, it is imperative to prioritize interventions and strategies aimed at addressing stunting to ensure the healthy growth and development of children in Addis Ababa.

In this study, significant factors associated with stunting in school-age children were identified. It was found that the marital status of parents/caregivers and house ownership were significant factors for stunting. It was also found that school-aged children whose parents/caregivers are single were more likely to experience stunted growth. This indicates that single parents/caregivers faced challenges in providing adequate nutrition and psychosocial stimulation. A previous study support this finding that single parents/caregivers often experience higher levels of stress and have less time and resources available to devote to their children compared to two-parent households (40).

The results indicate that school-aged children living in rented houses have a higher prevalence of stunting compared to those residing in privately owned homes. This finding suggests a potential link between housing stability and nutritional status. Additionally, it was noted that children in rented or government-owned houses often have limited access to essential water, sanitation, and hygiene facilities. In this regard, other studies confirmed that a lack of basic infrastructure in households leads to increased rates of waterborne diseases and poor hygiene practices (41). This could increase the risk of infections and malnutrition (42).

The educational level of the parents/caregivers was also found to be significant, with lower levels of education being associated with higher prevalence of stunting. With this perspective, the current study is consistent with similar research about the educational level of parents/caregivers and stunting in Ethiopia and Indonesia (43). Moreover, according to a WHO report from 2018, mothers with a lower level of education are more likely to have stunted children (44). Therefore, as it can be seen in the finding of this study, the education level of the parents can be considered as a factor for the prevalence of stunting among school-aged children, and this requires effective interventions aimed at improving parental education and awareness about nutrition and the importance of a balanced diet.

Concerning the school-aged children sex and stunting, the finding confirmed a significant association between school-aged children sex and stunting, with female children being less likely to suffer from stunting than male children. In addition, other researchers also confirmed that childhood stunting is consistently associated with male sex (45, 46); however, studies which were conducted in China and Pakistan showed different results; for instance, female children compared to male children were more likely to be stunted (47, 48). In this regard, stunted growth in male children can have long-term economic consequences for a country. Stunted children are more likely to experience developmental delays, cognitive impairment, and reduced physical capabilities, which can impact their productivity as adults (49). Therefore, investing in improving childhood nutrition is not only crucial for the wellbeing of individuals but also for the overall economic growth and development of a nation.

In this study, it was found that school-aged children who skipped meals were more likely to experience stunted growth. A study conducted in Egypt provides evidence that irregular meal times, skipping breakfast, and consuming fewer than three meals per day were correlated with stunted growth (50). Thus, this finding emphasizes the importance of regular and sufficient meals in preventing undernutrition and promoting healthy development in children. Therefore, efforts should be made at all levels to address the issue of chronic undernutrition in children, including providing access to nutritious food and promoting the importance of adequate meal times to ensure proper growth and development.

In this study, it was found that caregivers' nutritional knowledge had shown an impact on the risk of stunting in school-aged children. School-aged children whose parents/caregivers did not have awareness about nutrition had a higher risk of stunting. This indicates the importance of educating caregivers about nutrition and its impact on children's growth and development. In this regard, a study conducted in Tanzania emphasized the importance of nutrition education in influencing food quality and diversity in children (51). Therefore, it can be said that nutrition education programs should be implemented in communities and schools to raise awareness about proper nutrition and improve food quality and diversities among the community.

Regarding wasting among school-aged children, there was a prevalence rate of 15%. More than half of school-aged children in this study skipped over two mealtimes and this may contribute to the prevalence of wasting. In line with this, other research suggested that wasting could be a consequence of insufficient food intake (52). Compared to other studies, the result of this study is lower than the study conducted in the Gedeo Zone, South Ethiopia, which found a prevalence of 18.2%; however, higher than that of Gondar Town, northwest Ethiopia, with a prevalence of 9%−11% (53). Thus, it can be seen in the finding, there are a significant proportion of children who are undernourished and this may hinder children's growth and development, both physically and cognitively.

The other important findings in this study were factors linked to wasting in school-aged children. There was a statistically significant association between the wealth index (WI) and wasting among school-aged children. In line with this, the study conducted in other areas showed that household wealth index was significantly associated with wasting (54, 55). Families with lower economic status may struggle to afford an adequate diet, access healthcare services, or provide a hygienic living environment, all of which can contribute to a higher risk of malnutrition, including wasting, among children (56). Hence, addressing the underlying socioeconomic determinants of malnutrition, such as poverty alleviation, improving access to education, healthcare, and social safety nets, is essential to effectively combat wasting and other forms of malnutrition among school-aged children.

Furthermore, the prevalence of wasting was significantly lower for school-aged children aged 11–14 years compared to those aged 6–10 years. Concerning this, studies conducted in Gondar town, northwestern Ethiopia (35), and Gedeo Zone in South Ethiopia (51) showed the same result. Children between 11 and 14 years old are typically going through adolescence, a period of rapid growth and development (57). This phase requires higher nutritional intake compared to younger children. However, challenges such as inadequate diet, social pressures, and resource constraints can result in malnutrition, including wasting, and it will have profound consequences on an individual's health in later life, as well as the health of any potential children (58). From this, it can be suggested that focused interventions are needed to address the distinct nutritional requirements and challenges of this age group, which may involve educating them about healthy eating habits, ensuring access to nutritious foods, and tackling social and economic factors contributing to malnutrition.

Regarding the food groups that children consume, the finding shows that the school-aged children who consumed four or more food groups had lower prevalence of wasting. This finding is also supported by a similar study conducted in the Semien Bench district in Ethiopia (59). The implication of children who consumed more than four groups of foods associated with lower odds of wasting suggests a potential protective effect of dietary diversity against malnutrition, particularly wasting (60). Therefore, children who consume a diverse range of foods may have better access to essential nutrients necessary for growth, potentially reducing the risk of wasting. This finding highlights the importance of promoting dietary diversity and ensuring access to a wide range of nutritious foods to combat malnutrition among children.

The study also identified a significant link between dietary habits and wasting, which is supported by previous studies conducted in Tabriz, Iran (61), and the Abuja Municipal Area Council (62). Poor dietary habits, such as consuming a diet high in processed foods, sugar, and unhealthy fats while lacking essential nutrients like vitamins, minerals, and protein, can lead to malnutrition and wasting, compromising overall health and wellbeing (63). Thus, addressing this issue requires interventions focused on improving access to essential nutrients from vegetables and fruits, promoting education on healthy eating habits.

The other important factor identified in this study is that school-aged children who did not consume any absorption inhibitors (AI = No) had a 15% lower prevalence of wasting compared to those who consumed an absorption inhibitor (AI = Yes). This finding is consistent with that of a previous study conducted in Gondar town, northwest Ethiopia (35). Absorption inhibitors being associated with wasting suggests that factors interfering with the body's ability to absorb nutrients contribute to the development of wasting. Inhibitors such as phytates in tea and coffee can bind to minerals like iron and zinc, making them less available for absorption (64). Thus, addressing absorption inhibitors may involve improving dietary quality, or enhancing nutrient bioavailability through food processing or supplementation.

Furthermore, access to water supplies was associated with significantly lower prevalence of wasting, which is in agreement with studies conducted in rural Ethiopia (65) and Bangladesh. This finding highlights the multifaceted impacts of socioeconomic factors, dietary practices, and access to basic amenities on the prevalence of wasting among school-aged children. Access to clean water supplies and proper toilet facilities is essential for maintaining good hygiene practices, preventing waterborne diseases, and ensuring overall health and wellbeing (66). Hence, improving access to clean water supplies and sanitation facilities is critical for reducing the incidence of wasting and improving the nutritional status of populations.

The study found that 36% of school-aged children were underweight. This prevalence of underweight was higher than that reported in a previous study elsewhere in Ethiopia (41), but lower than that reported in studies from Gondar Zuria District (67) and Central India (68). This might be differences in study design, sample size, sampling methodology, data collection strategy, data quality, sociodemographic traits, food habits, degree of physical activity, and environmental factors can contribute to these discrepancies (69). Thus, addressing underweight prevalence often requires comprehensive interventions aimed at improving access to nutritious food, promoting healthy eating habits, enhancing healthcare services, addressing socioeconomic determinants of malnutrition, and implementing policies that support food security and nutritional wellbeing.

In this study, positive correlations between parental age and the risk of underweight among school aged children were identified. This finding is also consistent with a study conducted in Terengganu, Malaysia (70). In addition, children born to younger mothers had a lower likelihood of underweight. This might be due to the fact that younger mothers may be more likely to engage in healthier behaviors during pregnancy, such as eating a balanced diet and getting regular prenatal care. These factors contribute to better fetal growth and development (71). Hence, this finding highlights the importance of considering maternal characteristics, including age, in efforts to understand and address childhood undernutrition.

Moreover, the finding showed that children who had less than three mealtimes per day were more likely to be underweight. This highlights the significance of having regular and sufficient meals to prevent undernutrition. Among this sample, dietary changes are required to ensure that children follow a well-balanced diet for optimum health and development. This finding is further supported by research conducted in Bangladesh (72).

With regard to overweight, it was found that 19% of school-aged children were overweight, which implies most children in the study area are at risk of developing health complications related to excess weight. This finding is consistent with a study conducted in Kenya (73). However, it was higher than those of a previous study in Ethiopia reported by Kyallo et al. (74) and other developing countries, and Southern Nigeria (75). On the other hand, the finding was lower than the prevalence observed in Argentina (76) and Bangladesh (77). These differences could be attributed to variations in the methods of measuring and defining overweight, sample size and characteristics, socioeconomic and environmental factors, dietary and physical activity patterns, and genetic and biological factors among different populations (78).

The study also found a significant association between school type and being overweight among school-age children. Those attending public schools had a 56% lower chance of being overweight compared to their counterparts in private schools. This finding coincides with previous studies conducted in Dire Dawa in Eastern Ethiopia (79), Tanzania (74), and Kerman province, Iran (80). However, it was inconsistent with a study conducted in Spain (81), which showed that children from public schools were more likely to have excess weight than those from private schools. This discrepancy could be explained by different government funding for school feeding programs in high-income nations such as Spain and low-income nations such as Ethiopia.

## 5 Limitations of the study

This study had several limitations that should be acknowledged. These limitations include its cross-sectional design, reliance on self-reported data, and limited generalizability. However, despite these limitations, the study still provides valuable information for policymakers, healthcare professionals, and researchers. It reveals the factors that influence the nutritional status of school-age children and also offers recommendations for comprehensive, context-specific interventions.

## 6 Conclusions

Generally, there are high levels of both undernutrition and overnutrition among school-age children in urban low-income settings in Addis Ababa. The adjusted analysis identified key factors that influence stunting, wasting, underweight, and overweight, including parents' marital status, house ownership, education, child's gender, meal habits, and nutrition knowledge.

Interventions should target socioeconomic factors, parental education, and dietary practices. This study emphasizes the need for coordinated efforts by the government, scholars, and communities to address the complex nature of malnutrition. Furthermore, it suggests comprehensive nutrition education programs to empower parents and caregivers with the essential knowledge to provide adequate nutrition and psychosocial stimulation to their children, thereby mitigating the risk of stunting. Moreover, it recommends interventions aimed at improving housing conditions, particularly for families residing in rented or government-owned accommodations, which are crucial in enhancing access to water, sanitation, and hygiene facilities and can significantly reduce the susceptibility to infections and malnutrition among school-aged children.

## 7 Areas for further research

Our study's conclusions point to a number of areas that need more investigation to improve our knowledge of Addis Ababa's school-age population's nutritional status.

- Investigate how cultural beliefs and practices influence dietary choices and nutritional status among school-aged children, emphasizing the integration of these cultural dimensions into intervention programs.
- Assessing the availability and impact of community resources, such as food banks and nutrition centers, on the nutritional status of children in urban low-income settings.
- Evaluating the effectiveness of school feeding programs on improving the nutritional status of children in public vs. private schools could inform strategies for optimizing these programs for better outcomes.
- Conducting food environment assessments to evaluate the availability and accessibility of nutritious foods vs. processed foods would further illuminate how these factors influence dietary choices.

## Data Availability

The raw data supporting the conclusions of this article will be made available by the authors, without undue reservation.
